# Excessive Daytime Sleepiness and Its Associated Factors Among Male Road Transport Workers in Brazil

**DOI:** 10.1111/jsr.70198

**Published:** 2025-09-10

**Authors:** Renato Canevari Dutra da Silva, Maria Flávia Campos Adelino, André Furtado Duarte, Josiane Santos de Souza, Fábio Vieira de Andrade Borges, Carlabianca Cabral de Jesus Canevari, Anderson Garcez

**Affiliations:** ^1^ Faculty of Medicine and Dentistry University of Rio Verde, UniRV Rio Verde Brazil; ^2^ Post‐Graduate Program in Medical Sciences: Endocrinology Federal University of Rio Grande do Sul State, UFRGS Porto Alegre Brazil

**Keywords:** Brazil, excessive daytime sleepiness, sleep, truck driver

## Abstract

This study aimed to estimate the occurrence of excessive daytime sleepiness (EDS) and its associated factors among male road transport workers. A cross‐sectional study was conducted with a non‐probabilistic sample of 414 drivers recruited at gas stations and parking lots in Formosa and Rio Verde, Goiás, Brazil, in 2024. The presence of EDS was evaluated using the Epworth Sleepiness Scale, and the investigated associated factors included demographic, socioeconomic, behavioural, health and professional characteristics. Logistic regression was used to explore the factors associated with EDS. The prevalence of EDS in the sample was 39.9% (95% CI: 35.1–44.6). After adjustment, a higher probability of EDS was observed among drivers aged between 41 and 60 years, with non‐white skin colour, and those who were married. The analysis also indicated that drivers with high levels of anxiety and a high risk of obstructive sleep apnea were more susceptible to EDS whereas drivers with good sleep quality and adequate rest practices had a lower probability of EDS. Additionally, long working hours significantly increased the chance of EDS. In conclusion, the findings of this study revealed a high occurrence of EDS among male road transport drivers and its association with demographic characteristics, working conditions, mental health and sleep quality. Therefore, strategies addressing these factors are essential to reducing the occurrence of EDS and contributing to a safer road environment.

## 1 Introduction

Excessive daytime sleepiness (EDS) is an increasing concern among road transport workers, affecting both drivers' health and road safety. Previous studies have reported a high prevalence of EDS among drivers. For instance, a study involving 500 drivers found that approximately 30% experienced frequent episodes of daytime sleepiness, which was associated with factors such as irregular shifts and long working hours (AlShareef 2021). Another study supports these findings, emphasising that EDS is prevalent in professions characterised by unfavourable working hours (Wickwire et al. 2017).

The work environment and professional demands play a crucial role in the onset of sleepiness. Excessive workloads and insufficient breaks have been identified as significant contributors to an increased chance of EDS (Sá and Sampaio 2022). Drivers working more than 10 h per day are 2.5 times more likely to report sleepiness (Hege et al. 2015). The consequences of EDS extend beyond the individual, directly impacting occupational health. Previous studies have demonstrated an association between EDS and various health conditions, including sleep disorders, cardiovascular diseases and mental health disorders (Moradi et al. 2019; Pylkkonen et al. 2018). Drivers with EDS also exhibit a higher prevalence of hypertension and type 2 diabetes (Moradi et al. 2019). Moreover, daytime sleepiness has direct implications for traffic safety, as drowsy drivers are more prone to accidents (Pylkkonen et al. 2018).

In a sector where the safety of passengers and the integrity of cargo transportation are paramount, understanding the prevalence of EDS and its associations with professional characteristics is essential (Garbarino et al. 2016). In Goiás, Brazil, where road transportation plays a central role in mobility and logistics, the issue of daytime sleepiness is particularly critical due to extensive routes and challenging road conditions (Martins et al. 2017; Santos et al. 2013). This scenario underscores the urgency of addressing EDS not merely as an individual concern but as a public health issue that warrants further investigation. Therefore, this study aimed to estimate the occurrence of EDS and its associated factors among male road transport workers in Goiás, Brazil. Analysing this issue can provide valuable insights into drivers' working conditions and health, facilitating the identification of high‐risk groups and informing preventive strategies.

## 2 Methods

This was a cross‐sectional study contemplating a sample of 414 male drivers and conducted over three consecutive months (May–July) in the municipalities of Formosa‐GO and Rio Verde‐GO, Brazil, in 2024. The sample was non‐probabilistic by convenience, comprising all drivers present at the data collection sites during the 3 months. Data were collected at two gas stations along BR‐020 and at seed factory parking lots in Formosa, as well as at three gas stations along BR‐060. Formosa, located in southeastern Goiás, lies 280 km from Goiânia and 75 km from Brasília, covering an area of 5804.292 km^2^ with a population of approximately 125,705. Rio Verde spans 8379.7 km^2^ and is home to around 235,647 residents. This study was part of a broader project titled ‘Sleep‐Related Aspects of Transport Workers in Goiás and Their Relationship with Professional Characteristics and Health Interfaces’, approved by the Research Ethics Committee of the University of Rio Verde—UniRV, under opinion number 6.025.130, dated April 26, 2023.

The sample included truck drivers transporting any type of cargo, regardless of vehicle capacity, gender, or age. However, drivers with less than 6 months of uninterrupted experience were excluded (*n* = 23). Thus, a total of 431 questionnaires were collected, of which 414 were deemed valid for analysis. Seventeen questionnaires (3.9%) were excluded due to withdrawal or invalidation. Specifically, nine (52.9%) corresponded to participants who did not initiate completion, five (29.4%) were discontinued because of work‐related demands during working hours and three (17.7%) were invalidated owing to erasures that compromised response consistency. Only fully completed and valid questionnaires were included in the analysis, thereby ensuring data quality.

Participants were approached during rest and/or meal breaks, predominantly at night, in pre‐designated locations provided by the establishments, ensuring the confidentiality of the volunteers. The researchers provided the study instruments directly to the drivers, explaining the research procedures beforehand. After completion, the questionnaires were collected, organised in numbered folders according to the data collection period, and prepared for subsequent analysis and coding. Extraordinary meetings were held between researchers, students and the project team to standardise procedures.

The outcome variable was EDS, defined as an increased tendency to fall asleep in situations that others might consider inappropriate. This was chosen due to its consequences for individuals' quality of life and their performance in professional activities and traffic. To assess EDS, the Epworth Sleepiness Scale translated and validated for Brazilian Portuguese was used (Bertolazi et al. 2009), measuring the likelihood of dozing off in eight everyday situations, with scores ranging from 0 to 24. The presence of EDS was defined as an Epworth Sleepiness Scale score of 10 or higher (score ≥ 10) (Bertolazi et al. 2009; Johns 1991).

The investigated associated factors (exploratory variables) included demographic, socioeconomic, behavioural, health and professional characteristics. The demographic characteristics include age groups (< 40; 41–60; > 60 years), skin colour/race (white; non‐white) and marital status (married; unmarried). The socioeconomic characteristics encompass education level (with high school or higher education; without high school completed), and cohabitation (‘Who do you live with?’: with someone; alone). The behavioural characteristics include self‐reported leisure‐time physical activity (≥ 150 min per week: no; yes) (Mielke et al. 2021), number of daily meals at work (≤ 2; > 2); fast food consumption (rarely; frequently), soft drink consumption (rarely; frequently), candy consumption (rarely; frequently), coffee consumption (no; yes) and frequency of daily coffee consumption (no; up to three times; more than three times); alcohol consumption (‘Have you ever consumed alcoholic beverages?’—responses ‘no’ and ‘yes, but I no longer drink’ were grouped as ‘no,’ and the others as ‘yes’), current smoking tatus (‘Do you smoke or have you ever smoked manufactured cigarettes?’—responses ‘no’ and ‘yes, but I'm a former smoker’ were categorised as ‘no,’ and the others as ‘yes’), nicotine dependence (evaluated by the Fagerström Test) (Halty et al. 2002), and categorised as: no; moderate; high dependence, use of psychoactive substances (‘In the last 30 days, how many days did you use substances to stay awake?’—responses ‘I've never used such substances’ and ‘no days in the last 30 days’ were categorised as ‘no,’ and the others as ‘yes’), and illicit drug use (‘In the last 30 days, how many days did you use drugs such as marijuana, cocaine, crack and so on?’—responses ‘no days’ were categorised as ‘no’, and the others as ‘yes’).

The health characteristics included nutritional status, using the body mass index (BMI) obtained by self‐reported body weight and height and classified as: normal (< 25.0 kg/m^2^), overweight (25.0–29.9 kg/m^2^) and obesity (≥ 30.0 kg/m^2^) (WHO 2000). Additionally, associated diseases were evaluated (‘Has any doctor ever told you that you have or had a diagnosed disease?’—categorised as ‘no’ or ‘yes’), as well as mental‐health‐related variables including depression, anxiety and stress were assessed using the Depression, Anxiety and Stress Scale (DASS‐21) (Vignola and Tucci 2014), with low (normal and mild) or high‐risk (moderate, severe, extremely severe) categories, and psychological distress, assessed by the Kessler Psychological Distress Scale (K10) (Lins et al. 2021), categorised as low or high risk (Lins et al. 2021; Vasiliadis et al. 2015). Finally, sleep‐related variables were analysed using the Pittsburgh Sleep Quality Index (PSQI) (Bertolazi et al. 2011), categorising as satisfactory (PSQI ≤ 5) or unsatisfactory sleep (PSQI > 5) (Bertolazi et al. 2011) and the risk of obstructive sleep apnea syndrome (OSAS) was evaluated using the Berlin Questionnaire (Vaz et al. 2011), categorised as low or high risk (Silva et al. 2022).

The professional characteristics include years of experience in the profession (‘How long have you been working with truck transportation?’—categorised as: ≤ 20; 21–40; > 40 years), type of cargo transported (‘What is the most frequently transported cargo?’—categorised as: grains; fuel; dry cargo; food; others), kilometres driven per day (‘How many kilometres do you drive daily?’—categorised as: ≤ 500; 501–800; > 800 km), work shift (‘What is your work shift?’—categorised as morning/afternoon; morning/afternoon/night); others ([morning/night or afternoon/night]), hours worked per day (‘How many hours do you usually work per day?’—categorised as: ≤ 8; 9–12; > 12 h), and self‐reported involvement in accidents (no; yes).

Data entry was performed using the EpiData 3.1 software, with double entry to avoid typing errors. Data consistency and validation were ensured. Data analysis was performed using descriptive and inferential statistics, using the Jamovi v2.5.6 and SPSS v27 software. Descriptive measures were calculated, along with absolute (*n*) and percentage frequencies (%). First, a bivariate analysis was conducted to explore the factors associated with EDS, using the chi‐square test for heterogeneity of proportions. After, the odds ratios (OR) with 95% confidence intervals (CI) were estimated by binary logistic regression model (unadjusted analysis). Variables with a significance level of *p* < 0.30 in unadjusted analysis were simultaneously included in the multivariate analysis. Using the Backward method, variables that were not significant were removed, and only those with statistically significant associations (*p* < 0.05) remained in the final model.

## 3 Results

A total of 414 male road transport workers participated as volunteers, with a mean age of 46.68 ± 10.49 years. Tables 1 and 2 present the sample characteristics.

**TABLE 1:** Sample characteristics and prevalence of excessive daytime sleepiness (EDS) according to demographic, socioeconomic and behavioural characteristics among male road transport workers in Goiás, Brazil, 2024 (*N* = 414).

Characteristics	Total	EDS	
	*n* (%)	*n* (%)	*p* *
Age (years)			0.061
≤ 40	129 (31.2)	44 (34.1)	
41–60	245 (59.1)	109 (44.5)	
> 60	40 (9.7)	12 (30.0)	
Skin colour/race			0.001
White	239 (57.7)	54 (30.9)	
Non‐white	175 (42.3)	111 (46.4)	
Marital status			< 0.001
Unmarried	148 (35.7)	88 (33.1)	
Married	266 (35.7)	77 (52.0)	
Education level			0.499
With high school or higher education	179 (43.2)	68 (38.0)	
Without high school completed	235 (56.8)	97 (41.3)	
Cohabitation			0.118
Live with someone	364 (87.9)	140 (38.5)	
Live alone	50 (12.1)	25 (50.0)	
Physical activity (≥ 150 min/week)			0.268
No	325 (78.5)	125 (38.5)	
Yes	89 (21.5)	40 (44.9)	
Daily meals at work			0.858
≤ 2	213 (51.4)	84 (39.4)	
> 2	201 (48.6)	81 (40.3)	
Fast food consumption			0.750
Rarely	359 (86.7)	142 (39.6)	
Frequently	55 (13.3)	23 (41.8)	
Soft drink consumption			0.045
Rarely	289 (69.8)	106 (36.7)	
Frequently	125 (30.2)	59 (47.2)	
Candy consumption			0.140
Rarely	312 (75.4)	118 (37.8)	
Frequently	102 (24.6)	47 (46.1)	
Coffee consumption			0.001
No	32 (7.7)	4 (12.5)	
Yes	382 (92.3)	161 (42.1)	
Daily coffee consumption			0.002
None	32 (7.7)	4 (12.5)	
Up to three times	197 (47.6)	90 (45.7)	
More than three times	185 (44.7)	71 (38.4)	
Alcohol consumption			0.072
No	165 (39.9)	57 (34.5)	
Yes	249 (60.1)	108 (43.4)	
Current smoking status			0.250
No	311 (75.1)	119 (38.3)	
Yes	103 (24.9)	46 (44.7)	
Nicotine dependence (Fagerström test)			0.258
None	311 (75.1)	119 (38.3)	
Moderate	71 (17.2)	29 (40.8)	
High	32 (7.7)	17 (53.1)	
Psychoactive substance use (last 30 days)			0.476
No days	127 (30.7)	56 (44.1)	
1 to 5 days	12 (2.9)	4 (33.3)	
Does not use	275 (66.4)	105 (38.2)	
Illicit drug use			0.328
No	275 (66.4)	105 (38.2)	
Yes	139 (33.6)	60 (43.2)	

Abbreviations: 95% CI: 95% confidence interval; PR; prevalence ratio (reference category represented by PR = 1).

* Chi‐square test.

**TABLE 2:** Sample characteristics and prevalence of excessive daytime sleepiness (EDS) according to health and professional characteristics among male road transport workers in Goiás, Brazil, 2024 (*N* = 414).

Characteristics	Total	EDS	
	*n* (%)	*n* (%)	*p* *
Nutritional status (body mass index)			0.258
Normal (< 25.0 kg/m^2^)	89 (21.5)	29 (32.6)	
Overweight (25.0–29.9 kg/m^2^)	172 (41.5)	74 (43.0)	
Obesity (≥ 30.0 kg/m^2^)	153 (37.0)	62 (40.5)	
Associated diseases			< 0.001
No	226 (54.6)	70 (31.0)	
Yes	188 (45.4)	95 (50.5)	
Depression (DASS‐21)			0.030
Low risk	353 (85.3)	133 (37.7)	
High risk	61 (14.7)	32 (52.5)	
Anxiety (DASS‐21)			< 0.001
Low risk	324 (78.3)	112 (34.6)	
High risk	90 (21.7)	53 (58.9)	
Stress (DASS‐21)			< 0.001
Low risk	367 (88.6)	135 (36.8)	
High risk	47 (11.4)	30 (63.8)	
Psychological distress (K10)			< 0.001
Low risk	324 (78.3)	111 (34.3)	
High risk	90 (21.7)	54 (60.0)	
Sleep quality (PSQI)			0.001
Unsatisfactory sleep	224 (54.1)	92 (48.4)	
Satisfactory sleep	190 (45.9)	73 (32.6)	
Risk of OSAS			< 0.001
Low risk	247 (59.7)	80 (32.4)	
High risk	167 (40.3)	85 (50.9)	
Time in profession (years)			0.003
≤ 20	244 (58.9)	82 (33.6)	
21–40	147 (35.5)	75 (51.0)	
> 40	23 (5.6)	8 (34.8)	
Type of cargo transported			0.060
Grains	226 (54.6)	82 (36.3)	
Fuel	28 (6.7)	14 (50.0)	
Dry cargo	22 (5.3)	7 (31.8)	
Food	14 (3.4)	10 (71.4)	
Other	124 (30.0)	52 (41.9)	
Kilometres driven per day			0.264
≤ 500	103 (24.9)	46 (44.7)	
501–800	289 (69.8)	111 (38.4)	
> 800	22 (5.3)	8 (36.4)	
Work shift			< 0.001
Morning and afternoon	82 (19.8)	27 (32.9)	
Morning, afternoon and night	255 (61.6)	86 (33.7)	
Others (morning/night or afternoon/night)	77 (18.6)	52 (67.5)	
Hours worked per day			< 0.001
≤ 8	82 (19.8)	27 (32.9)	
9–12	254 (61.4)	86 (33.9)	
> 12	78 (18.8)	52 (66.7)	
Involvement in accidents			0.222
No	300 (72.5)	125 (41.7)	
Yes	114 (27.5)	40 (35.1)	

Abbreviations: 95% CI: 95% confidence interval; DASS‐21: Depression, Anxiety and Stress Scale; K10: Kessler Psychological Distress Scale; OSAS: obstructive sleep apnea syndrome; PR: prevalence ratio (reference category represented by PR = 1); PSQI: Pittsburgh Sleep Quality Index.

* Chi‐square test.

The sample consists exclusively of male workers (100%), with the majority identifying as non‐white (57.7%). Most are either single or separated (64.3%) and have not completed high school (56.8%). Alcohol consumption is prevalent, reported by 60.1% of workers, while 66.4% have never used drugs (Table 1). Regarding health conditions, 45.4% of workers reported having at least one associated disease, with diabetes mellitus and systemic hypertension being the most common, particularly among those aged 41 to 60 years (59.1%). In terms of BMI, 41.5% of workers had overweight, and 37% were classified with obesity. Physical activity levels are low, with 78.5% of workers failing to meet the minimum recommendation of 150 min of weekly exercise. Most workers consume coffee (92.3%), with 44.7% drinking it more than three times daily. Sleep quality is also a concern, as 45.9% report unsatisfactory sleep, and 40.3% are at high risk for OSAS. Regarding cargo transportation, grains are the most commonly transported (54.6%), followed by other types of cargo (30%) (Table 2).

The prevalence of EDS in the sample was 39.9% (95% CI: 35.1–44.6). The mean Epworth Sleepiness Scale score in the total sample was 8.96 (± 6.17; 95% CI: 8.37–9.56), with a mean of 15.87 (± 3.51; 95% CI: 15.33–16.41) among participants with EDS and 4.39 (± 1.58; 95% CI: 4.19–4.59) among those without EDS. Table 1 presents the prevalence of EDS based on demographic, socioeconomic and behavioural characteristics. Among demographic factors, EDS was more common in non‐white individuals (46.4%) compared to white individuals (30.9%). Regarding marital status, married drivers had a higher prevalence of EDS (52.0%) than unmarried drivers (33.1%). In terms of consumption habits, drivers who consumed coffee had a higher prevalence of EDS (42.1%) compared to those who did not (12.5%). Similarly, frequent soda consumption was associated with a higher prevalence of EDS, with 47.2% of regular soda consumers reporting EDS, compared to 36.7% of those who consumed it rarely (Table 1).

Table 2 presents EDS prevalence by health and professional characteristics. Drivers with health conditions had a higher prevalence of EDS (50.5%) than those without (31%). EDS was also more common among drivers at high risk for anxiety (58.9% vs. 34.6%), depression (52.5% vs. 37.7%), stress (63.8% vs. 36.8%) and psychological distress (60% vs. 34.3%). Unsatisfactory sleep was linked to higher EDS prevalence (48.4% vs. 32.6%), as was a high risk of obstructive sleep apnea (50.9% vs. 32.4%). Experience also influenced EDS, with drivers having 21–40 years of experience showing a higher prevalence (51.0%) than those with up to 20 years (33.6%). Shift work had a strong impact: drivers working two shifts had higher EDS prevalence (67.5%) than those working only mornings and afternoons (32.9%). Similarly, EDS was more frequent among those working over 12 h per day (66.7%) compared to those with shifts of up to 8 h (32.9%) (Table 2).

Table 3 presents the final logistic regression model for EDS prevalence, identifying nine statistically significant factors: age, skin colour, marital status, nutritional status, anxiety, sleep quality, chance of OSAS, daily kilometres driven and daily working hours. Drivers aged 41–60 years had a fivefold higher chance of EDS compared to those over 60 (OR = 5.44; 95% CI: 2.13–13.87), while those under 40 had nearly four times the chance (OR = 3.71; 95% CI: 1.36–10.10). Non‐white drivers had approximately four times the chance of EDS compared to white drivers (OR = 4.12; 95% CI: 2.39–7.08), and married drivers had a significantly higher chance than unmarried drivers (OR = 2.55; 95% CI: 1.51–4.29). Drivers with overweight had nearly three times the chance of EDS (OR = 2.93; 95% CI: 1.50–5.73), as did those with a high chance of anxiety (OR = 3.75; 95% CI: 2.11–6.67) and obstructive sleep apnea (OR = 2.32; 95% CI: 1.33–4.06). Conversely, satisfactory sleep quality was associated with a lower chance of EDS (OR = 0.60; 95% CI: 0.37–0.99). Driving 501–800 km/day (OR = 0.27; 95% CI: 0.15–0.50) or more than 800 km/day (OR = 0.20; 95% CI: 0.06–0.67) was linked to a lower chance of EDS. However, working over 12 h/day drastically increased the chance of EDS (OR = 9.44; 95% CI: 3.87–22.99) (Table 3).

**TABLE 3:** Multivariate analysis for the association between the prevalence of excessive daytime sleepiness (EDS) according to associated factors among male road transport workers in Goiás, Brazil, 2024 (*N* = 414).

Characteristics	EDS	
	Adjusted OR (95% CI)	*p*
Age (years)		
≤ 40	3.71 (1.36–10.10)	0.010
41–60	5.44 (2.13–13.87)	< 0.001
> 60	1	
Skin colour/race		
White	1	
Non‐white	4.12 (2.39–7.08)	< 0.001
Marital status		
Unmarried	1	
Married	2.55 (1.51–4.29)	< 0.001
Nutritional status (body mass index)		
Normal (< 25.0 kg/m^2^)	1	
Overweight (25.0–29.9 kg/m^2^)	2.93 (1.50–5.73)	0.002
Obesity (≥ 30.0 kg/m^2^)	1.13 (0.53–2.38)	0.758
Anxiety (DASS‐21)		
Low risk	1	
High risk	3.75 (2.11–6.67)	< 0.001
Sleep quality (PSQI)		
Unsatisfactory sleep	1	0.045
Satisfactory sleep	0.60 (0.37–0.99)	
Risk of OSAS		
Low risk	1	
High risk	2.32 (1.33–4.06)	0.003
Kilometres driven per day		
≤ 500	1	
501–800	0.27 (0.15–0.50)	< 0.001
> 800	0.20 (0.06–0.67)	0.009
Hours worked per day		
≤ 8	1	
9–12	0.96 (0.50–1.83)	0.899
> 12	9.44 (3.87–22.99)	< 0.001

Abbreviations: DASS‐21: Depression, Anxiety and Stress Scale; OSAS: obstructive sleep apnea syndrome; PSQI: Pittsburgh Sleep Quality Index.

## 4 Discussion

This study assessed the prevalence of EDS and its associated factors among road transport workers in Goiás, Brazil, with a focus on its implications for safety and occupational health. The findings revealed a high prevalence of EDS among male road transport drivers and its association with demographic characteristics, working conditions, mental health and sleep quality. These results highlight the need for increased attention to the physical and mental health of these professionals, particularly regarding sleep and psychological well‐being.

The prevalence of EDS identified in this study (39.9%; 95% CI: 35.1–44.6) is high and represents a significant concern for drivers' health and safety. This finding aligns with national and international studies that have also reported high EDS rates among professional drivers. For example, a study conducted with truck drivers in Brazil found a similar prevalence of 38%, indicating that EDS is a recurrent issue in this population (Santos et al. 2013). However, at the international level, the literature presents concerning findings regarding EDS among drivers. A study in the United States reported that approximately 33% of truck drivers frequently experienced daytime sleepiness (Bener et al. 2017). Likewise, a European survey found that 40% of road transport drivers were at risk of EDS, emphasising the role of long working hours and inadequate rest in the development of this condition (Hirshkowitz et al. 2015). Additionally, a high prevalence of EDS (33%) has also been reported in the general population (Jaussent et al. 2017).

The 41–60 age group exhibited a higher chance of EDS in this study, aligning with existing literature that suggests aging negatively affects sleep quality due to physiological changes and an increased prevalence of comorbidities, such as hypertension and diabetes mellitus—both commonly associated with EDS (Casagrande et al. 2022). In our study, hypertension and diabetes mellitus were the most prevalent conditions, particularly among drivers in this age range, which may contribute to the higher prevalence of EDS, as these conditions can directly impact sleep quality and daytime alertness. Additionally, prolonged exposure to occupational stressors in this age group may exacerbate sleep deprivation and drowsiness (Mao et al. 2023). The high prevalence of EDS among non‐white drivers raises concerns about racial health disparities, suggesting that limited access to healthcare and unfavourable working conditions may increase vulnerability. These findings indicate that EDS may be influenced by socioeconomic factors and environmental stressors. The biological plausibility of these associations may be explained by chronic stress, which has been linked to inflammation and sleep disturbances, further exacerbating EDS (Mao et al. 2023; Tomson et al. 2025). Furthermore, the significant association between marital status (married drivers) and EDS may be attributed to the interaction between family stress and professional demands. The challenge of balancing family responsibilities with work‐related pressures can increase emotional strain, leading to compromised sleep quality (Useche et al. 2018). Prior research suggests that inadequate social support contributes to sleep disturbances, indicating that drivers experiencing family tensions may have an increased chance of EDS (Garbarino et al. 2018).

The relationship between demographic factors and EDS has been extensively examined in the literature, emphasising the influence of variables such as skin colour, education level and socioeconomic conditions on sleep quality (Dutra da Silva et al. 2022; Patel et al. 2010). In our study, non‐white drivers had a higher chance of EDS, aligning with previous research that highlights disparities in healthcare access and living conditions, which can negatively affect sleep quality (Al‐Khalil et al. 2024). Additionally, educational attainment varied significantly by skin colour, with 34.1% of non‐white drivers not completing high school, compared to 22.7% of white drivers. This reinforces the role of socioeconomic and educational factors as social determinants of health, directly influencing EDS prevalence. Furthermore, the identification of married drivers as a higher‐risk group suggests that family responsibilities and work‐related demands may contribute to a cycle of stress and sleep deprivation, supporting findings from previous studies (Useche et al. 2018).

The finding that drivers reporting good sleep quality had a reduced chance of EDS suggests that interventions aimed at improving sleep quality could be an effective strategy for mitigating this issue. Research indicates that promoting adequate rest environments and educating drivers about sleep hygiene can help reduce daytime sleepiness (Casagrande et al. 2022). Additionally, the association between EDS and mental health, particularly the higher chance of EDS among drivers at high risk for anxiety, reinforces the close relationship between psychological disorders and sleep quality. Anxiety can induce hypervigilance and difficulty relaxing, creating a vicious cycle of poor sleep and worsening mental health (Garbarino et al. 2018).

This study also highlights the strong relationship between EDS and OSAS, as drivers at high risk for OSAS were more than three times as likely to experience EDS. OSAS, characterised by respiratory interruptions during sleep, leads to sleep fragmentation and, consequently, daytime sleepiness (Guglielmi et al. 2018; Mao et al. 2023). Similarly, overweight drivers had a significantly higher chance of EDS, being nearly three times more likely to report it than those with a normal BMI. This finding reinforces the well‐established link between excess weight and sleep disorders, particularly OSAS, which is commonly observed in overweight individuals due to adipose tissue deposition in the upper airways, contributing to airway collapse during sleep and subsequent daytime sleepiness (Peppard et al. 2013). On the other hand, although obesity was associated with a high prevalence of EDS (40.5%), the relationship was not statistically significant. This result may be influenced by individual variations, sample size, or potential biases, warranting further investigation. Nevertheless, obesity remains a critical factor in negative sleep‐related outcomes, particularly in populations with high metabolic and cardiovascular risk (Young et al. 2008). Therefore, the identification and appropriate treatment of OSAS are essential, as this condition not only affects sleep quality but also increases the risk of traffic accidents.

The high prevalence of EDS observed in our study can be attributed to multiple factors, particularly professional characteristics. The nature of driving work often requires long hours, leading to sleep deprivation and accumulated fatigue. Studies indicate that drivers working more than 12 h per day have a significantly increased chance of EDS, consistent with our findings (Mao et al. 2023; Tomson et al. 2025). Additionally, exposure to occupational stressors, such as tight deadlines and extended shifts, can exacerbate daytime sleepiness, making this a critical public health issue that requires urgent attention. Another relevant factor is the relationship between mileage driven and EDS. Drivers covering 501–800 km per day exhibited a lower chance of EDS, which may be attributed to better rest practices or more structured working conditions (Garbarino et al. 2018). Conversely, drivers covering shorter distances may experience greater pressure to complete multiple trips within a single day, leading to increased fatigue and sleep deprivation. Moreover, the association between long working hours (over 12 h per day) and EDS is well supported by studies demonstrating the adverse effects of sleep deprivation on cognitive performance and attentional capacity (Guglielmi et al. 2018). Prolonged work without adequate rest can induce physiological changes, including hormonal dysregulation and oxidative stress, further exacerbating daytime sleepiness (Mao et al. 2023).

In summary, the findings of this study are well‐grounded in a plausible biological framework, reflecting the complex interaction between sociodemographic factors, mental health, sleep disturbances and working conditions. Understanding these relationships is essential for developing effective interventions and promoting the health and safety of drivers. This study has several strengths. We used a validated instrument applicable to the Brazilian population to assess EDS (Bertolazi et al. 2009; Johns 1991). Additionally, an adjusted multivariate analysis was conducted to explore EDS occurrence and its associated factors, incorporating multiple relevant characteristics into the analysis. However, our findings should be interpreted considering certain limitations. This study examined a non‐probabilistic sample of Brazilian male drivers, which necessitates caution when making inferences about other population groups. Furthermore, as a cross‐sectional study, the possibility of reverse causality cannot be ruled out, making it impossible to establish temporal relationships between variables. Although we examined the association between EDS and driving accident risk, it is important to note that the Epworth Sleepiness Scale is not an appropriate instrument for assessing sleep‐related driving risk. More specific tools, such as the recently developed Bordeaux Sleepiness Scale (BOSS) (Philip et al. 2023), may provide a more accurate approach to evaluating this relationship. Another limitation is that clinical variables were estimated using validated instruments; however, these tools do not provide definitive diagnoses. Finally, a potential limitation is that this study represents a sub‐study of a larger project; however, the broader project was specifically designed to investigate sleep‐related aspects among transport workers, which reinforces the relevance of the present findings.

## 5 Conclusions

In this study, we identified a high prevalence of EDS among road transport workers in the state of Goiás, Brazil. Our findings indicate that the primary factors associated with EDS include key demographic characteristics, working conditions, mental health and sleep quality. Specifically, age, skin colour and marital status were significantly associated with EDS, suggesting that sociodemographic factors influence drivers' health and reflect existing inequalities that should be addressed through health policies. Furthermore, drivers with high anxiety levels and poor sleep quality were more susceptible to sleepiness, highlighting the interplay between mental health and physical well‐being. These findings underscore the importance of a multidimensional approach to addressing EDS and inform public health interventions aimed at preventing EDS in this population.

## Author Contributions

**Renato Canevari Dutra da Silva:** conceptualization, methodology, writing – original draft, writing – review and editing, project administration, formal analysis. **Maria Flávia Campos Adelino:** conceptualization, methodology, writing – original draft, writing – review and editing. **André Furtado Duarte:** conceptualization, methodology, writing – original draft, writing – review and editing. **Josiane Santos de Souza:** conceptualization, methodology, writing – original draft, writing – review and editing. **Fábio Vieira de Andrade Borges:** conceptualization, methodology, writing – original draft, writing – review and editing. **Carlabianca Cabral de Jesus Canevari:** conceptualization, methodology, writing – original draft, writing – review and editing. **Anderson Garcez:** conceptualization, methodology, writing – review and editing, writing – original draft.

## Conflicts of Interest

The authors declare no conflicts of interest.

## Data Availability

The data that support the findings of this study are available from the corresponding author upon reasonable request.
